# A Case of Pancreatic Metastasis of Renal Cancer Coexisting With a Pancreatic Neuroendocrine Tumor in a Patient Diagnosed With Fulminant Type 1 Diabetes Mellitus

**DOI:** 10.7759/cureus.79489

**Published:** 2025-02-23

**Authors:** Kento Miyazaki, Masaharu Ishida, Masahiro Iseki, Masamichi Mizuma, Michiaki Unno

**Affiliations:** 1 Department of Surgery, Tohoku University, Sendai, JPN

**Keywords:** distal pancreatectomy, fulminant type 1 diabetes, pancreatic metastasis, pancreatic neuroendocrine tumor, renal cancer

## Abstract

Fulminant type 1 diabetes mellitus (F1DM) is a subtype of type 1 diabetes that typically arises from viral infections or exposure to anticancer drugs. In the absence of these triggers, F1DM rarely develops concurrently with pancreatic tumors. This report presents a unique case of F1DM occurring alongside a pancreatic neuroendocrine tumor (PanNET) and pancreatic metastasis of renal cancer. A 72-year-old man with a history of right nephrectomy for renal cancer 20 years prior presented with a sudden onset of severe thirst. Laboratory investigations revealed hyperglycemia, ketoacidosis, and depleted insulin levels, leading to a diagnosis of F1DM. Abdominal computed tomography imaging identified multiple, rapidly enhancing tumors within the pancreatic body and tail, suggestive of metastatic renal cancer. The patient underwent a distal pancreatectomy and splenectomy. Pathological examination confirmed the presence of metastatic renal cell carcinoma and neuroendocrine tumors within the pancreas. A significant reduction in the number of pancreatic islets was observed, and the remaining islets exhibited a complete absence of insulin production. The postoperative course was uneventful, with a notable decrease in the required insulin dosage by approximately 50%. We experienced a rare case of PanNET and pancreatic metastasis of renal cancer complicated with F1DM, in the absence of chemotherapy or viral infection. The possibility of a coexisting pancreatic tumor should be considered in cases of fulminant type 1 diabetes.

## Introduction

Fulminant type 1 diabetes mellitus (F1DM) is a subtype of type 1 DM characterized by sudden onset with ketoacidosis, prominent hyperglycemia, and impairment of insulin secretion [1]. F1DM has been reported to be associated with genetic predisposition, viral infection, and anticancer drugs [2]. The pathogenesis of F1DM, which can be triggered by immune checkpoint inhibitors, is considered to involve cytokine-induced apoptosis as one of the causes of beta cell destruction, but the details remain unclear [2]. However, there is no report that suggests a direct association between the disease and neoplasms. We report a case of pancreatic neuroendocrine tumor (PanNET) and pancreatic metastasis of renal cancer presenting as F1DM. According to the literature, 19.4% of acute-onset type 1 diabetes cases are classified as F1DM [3], the incidence of PanNET is estimated to be one per 100,000 individuals [4], and metachronous pancreatic metastasis from renal cell carcinoma is rare [5]. The coexistence of all three conditions is considered extremely rare.

## Case presentation

A 72-year-old male presented to a local clinic complaining of sudden thirst and increased drinking water consumption. Before visiting the hospital, the patient had no symptoms suggesting a viral infection. He underwent right nephrectomy for right renal cell carcinoma with a tumor thrombus in the inferior vena cava (T3N0M0) 20 years ago and had no family history of malignant tumors or DM. A blood test indicated elevated blood glucose and ketoacidosis, which suggested type 1 DM, and he was referred to our hospital. The laboratory data are shown in Table 1. Ketoacidosis was successfully corrected with intravenous saline infusion and insulin administration. Subsequently, a regimen of intensive insulin therapy was implemented, leading to the achievement of stable glycemic control.

**Table 1:** Laboratory data on admission ALT: alanine aminotransferase, AST: serum aspartate aminotransferase, BUN: blood urea nitrogen, HGB: hemoglobin, HCT: hematopoietic cell transplantation, RBC: red blood cell, WBC: white blood cell, γGTP: gamma-glutamyl transpeptidase

Variable (unit)	Result	Reference range
WBC (x10^3^/μL)	5.5	3.3-8.6
RBC (x10^6^/μL)	4.40	4.35-5.55
HGB (g/dL)	14.3	13.7-16.8
HCT (%)	44	40.7-50.1
Platelets (x10^3^/μL)	182	158-348
Total protein (g/dL)	6.5	6.6-8.1
Albumin (g/dL)	4	4.1-5.1
AST (U/L)	15	13-30
ALT (U/L)	15	10-42
γGTP (U/L)	15	13-64
Total bilirubin (mg/dL)	0.9	0.4-1.5
BUN (mg/dL)	23	8-20
Creatinine (mg/dL)	1.31	0.65-1.07
Sodium (mmol/L)	145	138-145
Potassium (mmol/L)	4.2	3.6-4.8
Chloride (mmol/L)	107	101-108
Amylase (U/L)	214	44-132
Lipase (U/L)	312	6-48
CRP (mg/dL)	0.99	0.00-0.14
Elastase-I (ng/dL)	1077	0-300
IgG4 (mg/dL)	6	11-121
Blood sugar (mg/dL)	424	73-109
HbA1c (%)	7.6	0.0-6.4
Anti-glutamic acid decarboxylase (GAD) antibody	negative	N/A
Anti-insulinoma-associated protein 2 (IA-2) antibody	negative	N/A
Anti-insulin	negative	N/A
Glucagon (pg/mL)	135	70-174
Gastrin (pg/mL)	83	42-200
Serum C-peptide immunoreactivity (ng/dL)	0.02	0.80-2.50
Urine C-peptide immunoreactivity (μg/day)	0.86	22.80-155.20
Serum ketone (μmol/L)	8881	28-120
Acetoacetic acid (μmol/L)	2436	14-68
3-hydroxybutyric acid (μmol/L)	6445	0-74
Blood gas analysis		
pH	7.212	7.35-7.45
pCO_2_ (mmHg)	32	90-100
pO_2_ (mmHg)	84.9	35-45
HCO_3_ (mEq/L)	12.4	22-26
Base excess (mEq/L)	-14.4	(-2) - (+2)

**Figure 1:**
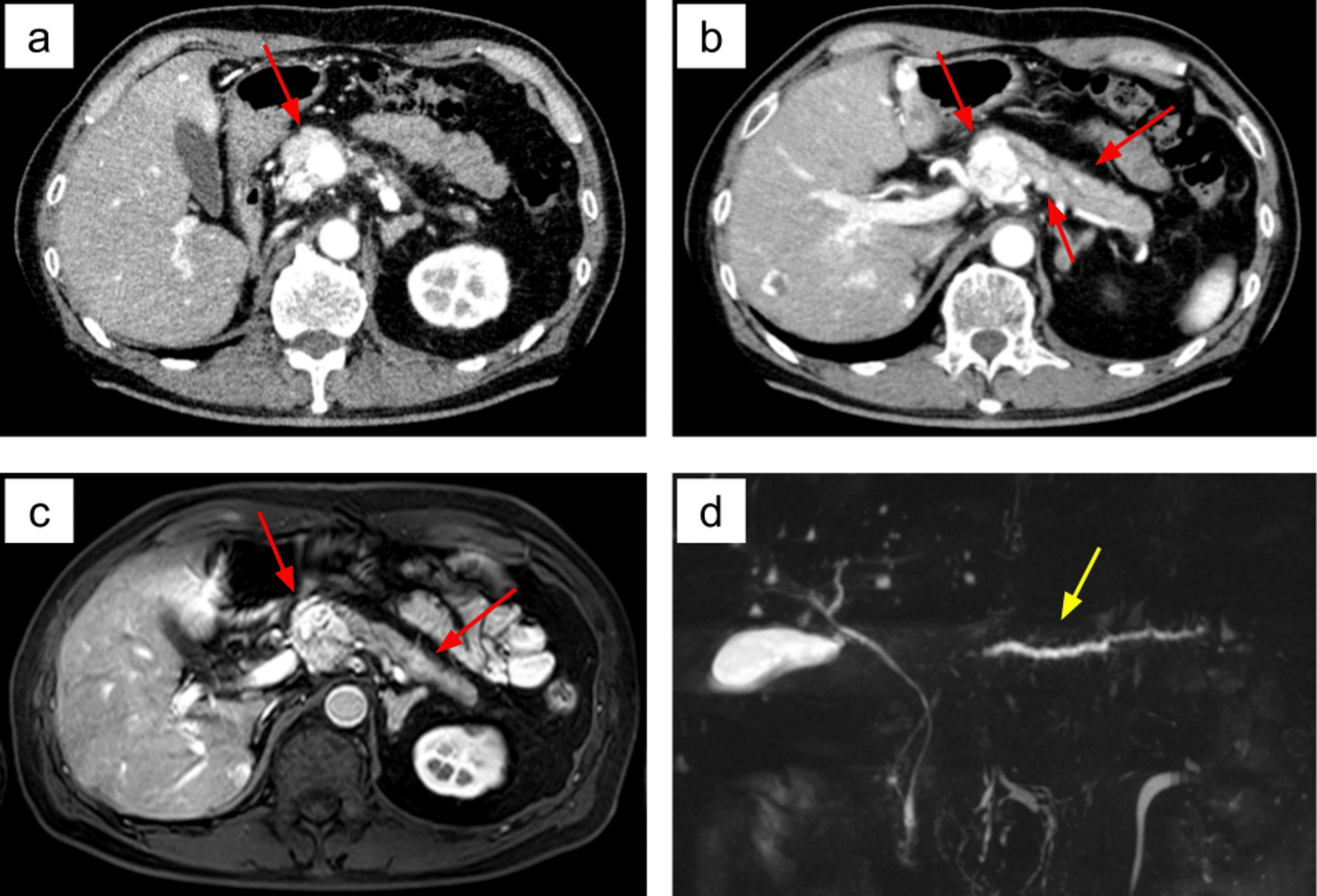
Preoperative images of the pancreatic tumors CT (a, b) and MRI (c, d) showed multiple hypervascular, early-enhancing tumors in the pancreas (red arrow). The largest of these, measuring 37 mm, was located in the pancreatic head (a, b, c). Smaller, intensely enhancing lesions were also present in the body and tail (b, c). MRCP demonstrated mild dilation of the main pancreatic duct in the body and tail (yellow arrow).

He was diagnosed with F1DM [3] based on the following findings: high blood glucose, relatively low HbA1c compared to the degree of hyperglycemia, reduced C-peptide, and rapid development of ketoacidosis within days of experiencing sudden thirst. Anti-islet antibodies, including anti-glutamic acid decarboxylase (GAD) antibody, anti-insulinoma-associated protein 2 (IA-2) antibody, and anti-insulin autoantibody (IAA), were negative, and pancreatic enzymes were elevated, which were findings consistent with F1DM [1]. Abdominal CT and MRI revealed multiple early-enhancing tumors in the pancreatic body and tail (Figure 1).

Fine needle aspiration cytology (FNA) was not performed to avoid bleeding of highly vascular tumors. We suspected pancreatic metastases of renal cancer based on his history of renal cancer. Pancreatectomy was planned because the metastases were limited in the pancreas, and no other distant metastases were observed in the imaging studies, including chest CT, FDG-PET, bone scintigraphy, and brain MRI. Blood glucose was controlled by insulin administration before surgery. Distal pancreatectomy and splenectomy were performed via laparotomy two months after the onset of symptoms, with a diagnosis of pancreatic metastases of renal cancer. The pancreatic tail showed marked lymphocytic infiltration with fibrosis and atrophy of pancreatic acinar tissue, which was suspected to be caused by the renal metastasis that occluded the main pancreatic duct. The pancreatic parenchyma was preserved in the pancreatic body.

**Figure 2:**
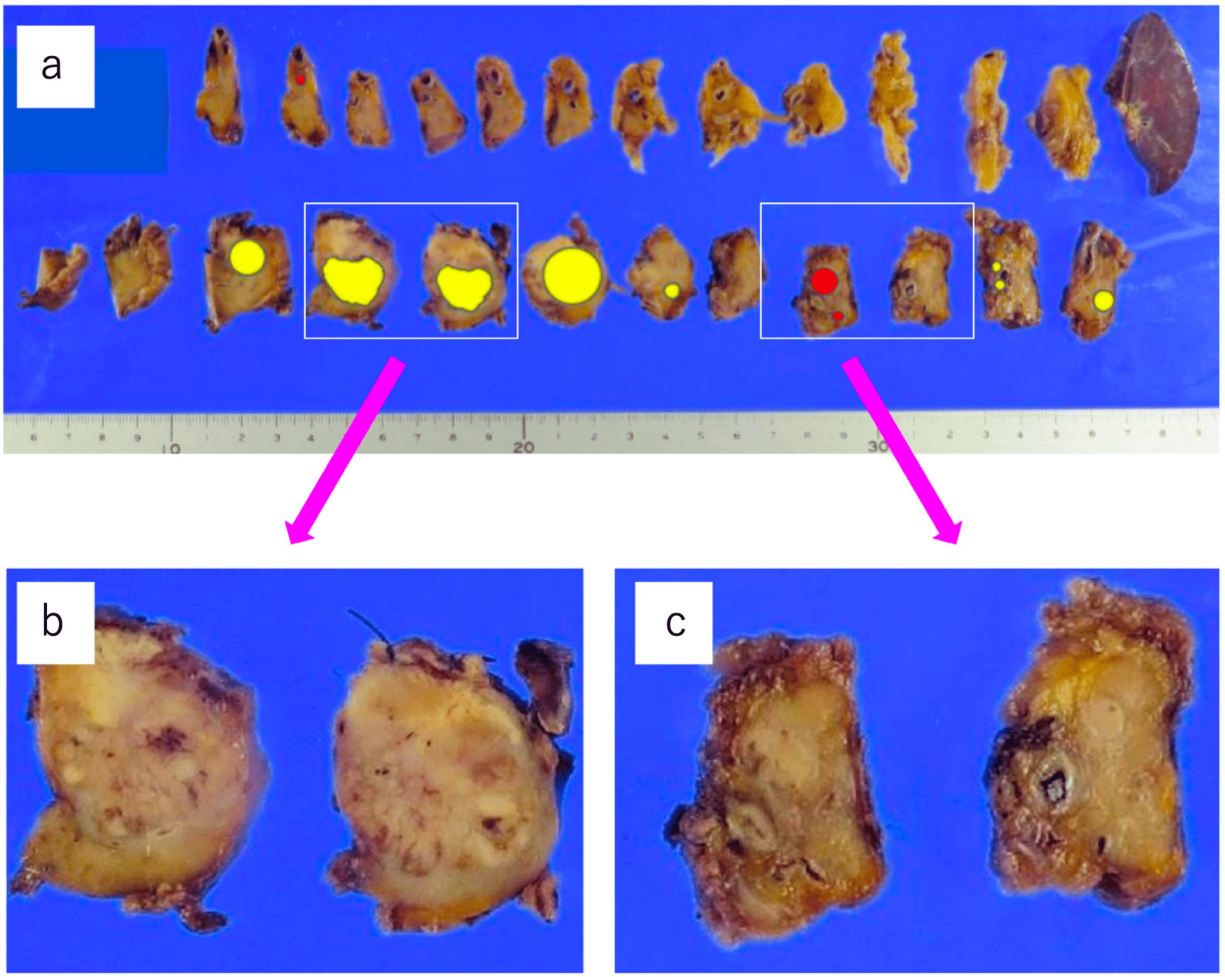
Macroscopic view of the surgical specimen a: The specimen was sliced sagittally after fixation. The yellow area corresponds to the lesion of pancreatic metastasis of renal cancer, and the red to PanNET. b: Close-up view of the 20-mm tumor in the pancreatic body. c: Close-up view of the 10-mm tumor in the pancreatic body.

Under macroscopic observation, there were multiple tumors in the pancreatic body and tail (Figure 2).

**Figure 3:**
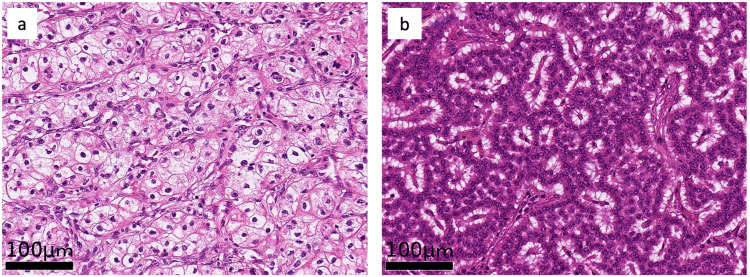
Hematoxylin-eosin staining of the pancreatic tumors a: The 20-mm tumor of the pancreatic body showed clear, acidophilic cell bodies and was diagnosed as pancreatic metastasis of renal cancer. b: The 10-mm tumor of the pancreatic tail showed oval, acidophilic cell bodies with funicular and ribbon-shaped proliferation and was diagnosed as PanNET.

The preoperatively recognized largest tumor of the pancreatic body (23 mm in size) showed a clear and acidophilic cell body and was diagnosed as metastasis of clear cell carcinoma (Figure 3).

**Figure 4:**
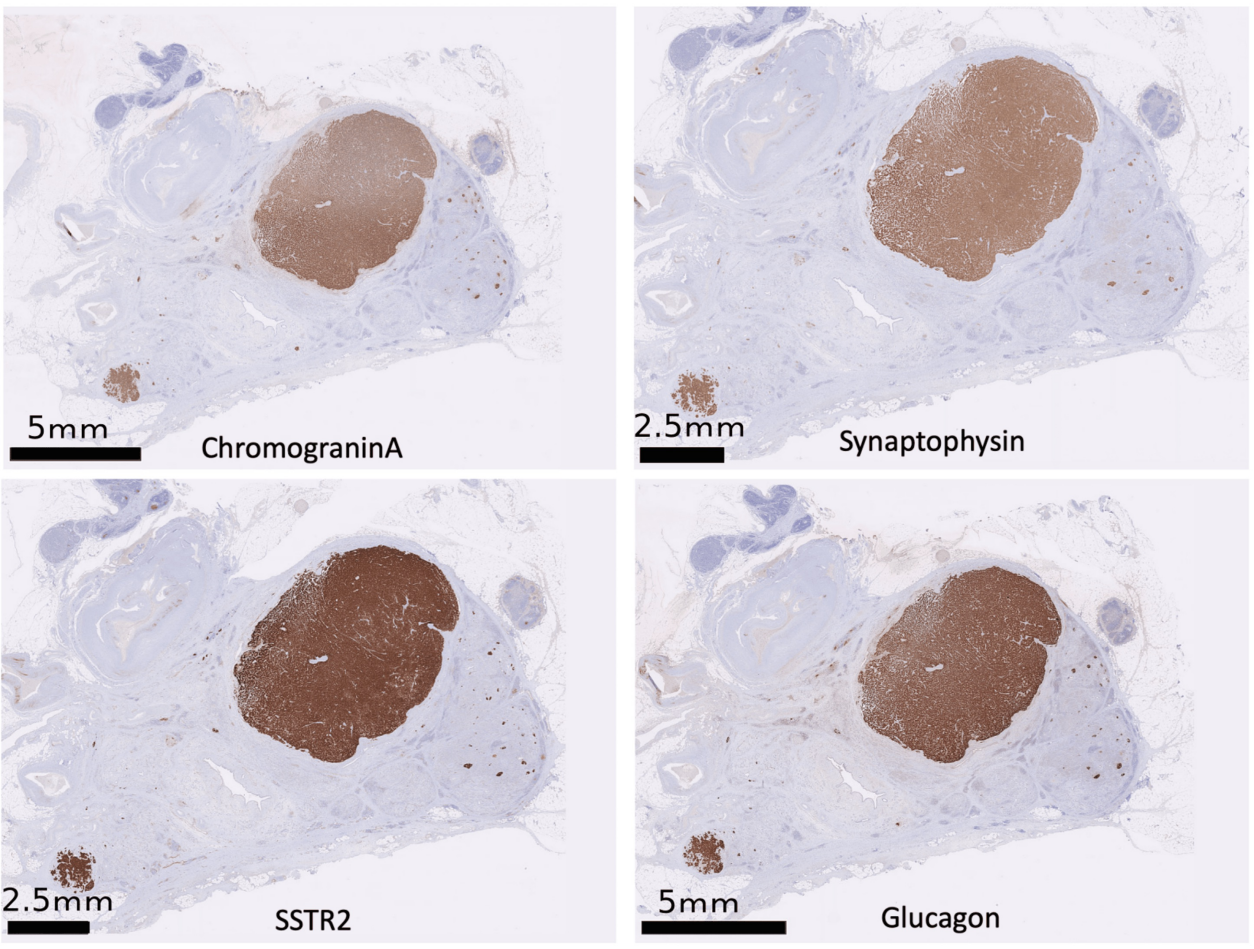
Immunohistochemistry of pancreatic neuroendocrine tumor (PanNET) The cells of PanNET were positive for chromogranin A, synaptophysin, somatostatin receptor 2 (SSTR2), and glucagon. These four biological substances were positive for PanNET.

The second largest tumor of the tail (8 mm in size) showed an oval and acidophilic cell body with funicular and ribbon-shaped proliferation and was diagnosed as a pancreatic neuroendocrine tumor (Figure 4).

**Figure 5:**
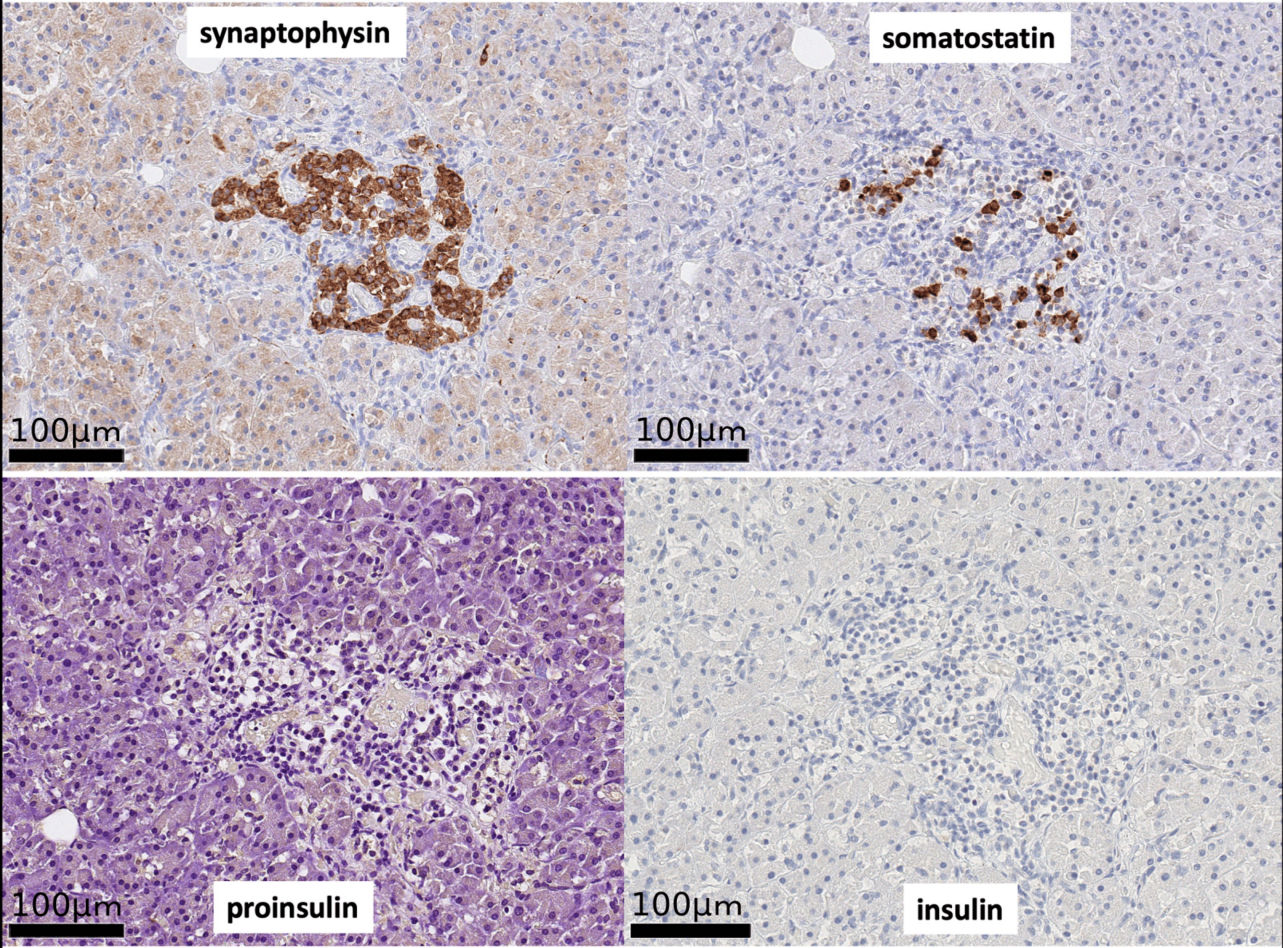
Immunohistochemistry of the remaining pancreatic islet The cells of the pancreatic islet were negative for insulin and proinsulin, while they were positive for synaptophysin and somatostatin.

Microscopic examination revealed four more tumors than radiologically identified. The PanNETs were positive for chromogranin A, synaptophysin, and glucagon, focally positive for gastrin, and negative for insulin, serotonin, and somatostatin. They demonstrated a Ki67 index of 1% and were classified into grade 1 (well-differentiated) according to the WHO classification (Figure 4). Pancreatic islets were scarcely found in the pancreatic parenchyma, and the cells in the remaining islets were negative for insulin (Figure 5).

The postoperative course was uneventful, and he was discharged on the 17th postoperative day. The amount of injected insulin decreased from 52 units per day before surgery to 26 units per day six months after surgery, whereas C-peptide remained low (0.01 ng/mL). Five years after the surgery, a total pancreatectomy was performed for pancreatic metastasis from renal cancer in the head of the pancreas.

## Discussion

According to the pattern of onset, type 1 DM is classified into three subtypes: fulminant, acute onset, and slowly progressive. F1DM is characterized by abrupt onset and near-complete depletion of insulin secretion at an early stage [1].

The disease is suspected to be caused by the destruction of β-cells resulting from the immunologic response influenced by viral infection or anti-tumor drugs, particularly immune checkpoint inhibitors, under certain genetic predispositions. A number of patients with F1DM have a history of preceding infection with several viruses, such as enterovirus, rotavirus, cytomegalovirus, Epstein-Barr virus, human herpesvirus 6, or others [1,3,6]. Genetic mutations associated with this disease are single-nucleotide polymorphisms in the human leukocyte antigen region, especially in the class II DR region [7], cytotoxic T-lymphocyte antigen 4 (CTLA-4) [8], and CSAD/Inc-ITGB7-1, which encodes cysteine sulfinic acid decarboxylase [9,10]. Immune checkpoint inhibitors, such as CTLA-4 antibodies (ipilimumab and tremelimumab), PD-1 antibodies (nivolumab and pembrolizumab), and PD-L1 antibodies (atezolizumab, avelumab and durvalumab), are known to induce F1DM as immune-related adverse events. Hosokawa et al. hypothesized that the suppression of immune regulators promotes T cells to attack not only cancer cells but also β-cells [2].

Because the patient had no history of administration of anti-tumor drugs, viral infections, or even suspected symptoms, the cause of F1DM is unknown. We proposed two hypothetical mechanisms for this case. First, pancreatitis induced by occlusion of the main pancreatic duct by the tumors caused F1DM, because pancreatitis itself could be the cause of F1DM [11]. Second, immune reactions to pancreatic tumors might have directly impaired β-cells, inducing F1DM. However, it seems very rare, as no association between F1DM and neoplasm has yet been reported. The tumors might have a connection to the pathophysiology of F1DM in this patient because the amount of injected insulin decreased to almost half after pancreatectomy. Although hormonal testing did not reveal a clear cause, we suspect that glucagon secretion from a neuroendocrine tumor may have contributed to the higher preoperative insulin requirement. In general, 35-70% of type 1 diabetes patients experience a period where they temporarily reduce the amount of insulin, called the “honeymoon period” [12]. However, our case is unlikely to be in that period because the reduced amount of insulin has been maintained after five years of surgery. To the best of our knowledge, this report is the first case of F1DM associated with pancreatic neoplasm without chemotherapy or viral infection.

## Conclusions

We experienced a rare case of PanNET and pancreatic metastasis of renal cancer complicated with F1DM, in the absence of chemotherapy or viral infection. The possibility of a coexisting pancreatic tumor should be considered in cases of F1DM.
